# A binding cooperativity switch driven by synergistic structural swelling of an osmo-regulatory protein pair

**DOI:** 10.1038/s41467-019-10002-9

**Published:** 2019-04-30

**Authors:** Abhishek Narayan, Soundhararajan Gopi, David Fushman, Athi N. Naganathan

**Affiliations:** 1 Department of Biotechnology, Bhupat & Jyoti Mehta School of Biosciences, Indian Institute of Technology Madras, Chennai, 600036 India; 2 Center for Biomolecular Structure and Organization, Department of Chemistry & Biochemistry, University of Maryland, College Park, MD 20742 USA

**Keywords:** Biochemistry, Biophysical methods, Optical spectroscopy, Biophysics, Computational biophysics

## Abstract

Uropathogenic *E. coli* experience a wide range of osmolarity conditions before and after successful infection. Stress-responsive regulatory proteins in bacteria, particularly proteins of the Hha family and H-NS, a transcription repressor, sense such osmolarity changes and regulate transcription through unknown mechanisms. Here we use an array of experimental probes complemented by molecular simulations to show that Cnu, a member of the Hha protein family, acts as an exquisite molecular sensor of solvent ionic strength. The osmosensory behavior of Cnu involves a fine-tuned modulation of disorder in the fourth helix and the three-dimensional structure in a graded manner. Order-disorder transitions in H-NS act synergistically with molecular swelling of Cnu contributing to a salt-driven switch in binding cooperativity. Thus, sensitivity to ambient conditions can be imprinted at the molecular level by tuning not just the degree of order in the protein conformational ensemble but also through population redistributions of higher-order molecular complexes.

## Abstract (web summary)

The bacterial protein Cnu together with the transcription repressor H-NS regulate expression of virulence factors in an osmo-sensitive manner. Here authors show that the structure of Cnu swells with decreasing ionic strength driving the oligomerization of H-NS and regulating osmo-sensory response.

## Introduction

Bacterial systems have evolved numerous regulatory mechanisms to control the expression of both virulence and non-virulence genes in response to external environmental variables^1–5^. Such regulatory mechanisms enable the survival of bacteria under diverse conditions be it in soil or upon infection of a host. It is well known that an osmotic shock involving a hypertonic solution induces an immediate shrinking of *E. coli* cells; this is followed by increased expression of specific genes that in turn modify the proteome up to nearly 30 min or longer^6,7^. Numerous outer-membrane proteins, aquaporins, and enzymes are over-expressed enabling accumulation of solutes either through active transport or synthesis to maintain osmotic balance and hence growth^5,8,9^. The reverse happens in a hypotonic solution or a low osmolarity environment wherein the cell volume increases, followed by efflux of ions and solutes. These reversible changes modulate the cellular proteome, crowding effects, and the amount of biologically active (or free) water thus controlling and contributing to protein conformational changes in the native ensemble, (un)folding, and oligomerization^10–14^.

In pathogenic bacteria, apart from temperature, osmolarity changes signal successful invasion of a mammalian host. For example, it has been observed that environmental osmolarity is a key regulator of the expression of *Leptospira sp*. (causes jaundice and renal failure in humans) immunoglobulin-like repeat proteins that are associated with virulence^15^. Similarly, uropathogenic *E. coli* (UPEC), the main causative agent of urinary tract infection in humans, experience dramatic changes in external osmolarity that range from ~0 Osm in soil to 1 Osm (~0.5 M ionic strength) upon infection^12,16,17^. The Hha-family proteins, all of which are single-gene products, have been implicated in regulating the expression of α-haemolysin in an osmo-sensitive manner thus contributing to virulence^1,18–20^. Hha-family proteins themselves do not bind DNA, but control the binding affinity and oligomerization of a transcription repressor H-NS on DNA. The complex between Hha-family proteins and H-NS also silences newly acquired genes contributing to antibiotic resistance and effectively playing an important role in environmental sensing^20–29^.

**Fig. 1:**
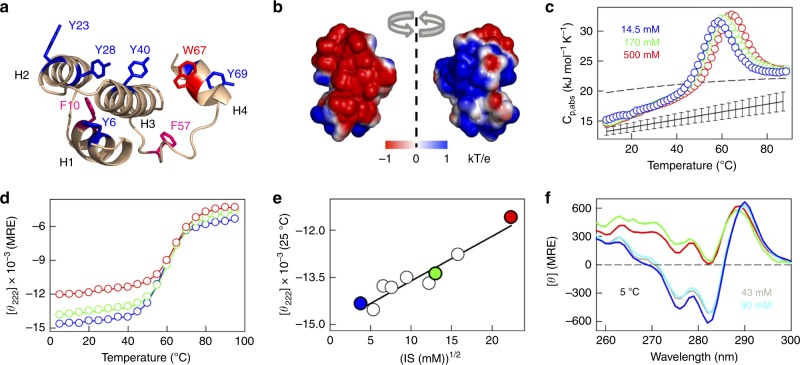
Breakdown of structure-stability relationship. **a** The structure of Cnu indicating the four helices and aromatic residues. **b** Electrostatic potential map of Cnu highlighting the large asymmetry in charge distribution. **c**, **d** Absolute heat capacity profiles (**c**) and the far-UV CD monitored thermal unfolding curves (**d**) at the representative ionic strength conditions. **e** The degree of secondary structure (ordinate) as a function of ionic strength at 25 °C (abscissa). The three representative conditions are marked in blue (14.5 mM), green (170 mM), and red (500 mM), respectively. Additional spectra were acquired at various intermediate ionic strengths spanning the representative conditions (open circles). **f** Near-UV CD spectra at 5 °C display significant tertiary structural differences at low ionic strength (<100 mM) compared to high IS conditions. Source data are provided as a Source Data file

One of the members of the Hha-family of proteins is Cnu (also referred to as YdgT), a four-helix bundle protein with a unique topology (Fig. 1a)^30^. As with other members of the Hha-family^31^, Cnu exhibits an asymmetric partitioning of charged residues on its surface: a large negative electrostatic potential on the H-NS binding face and a positive/neutral electrostatic potential on the opposite face (Fig. 1b). Cnu is however distinct from other members of the Hha-family in having a longer disordered loop connecting the third and fourth helices^32^. This longer loop promotes progressive unfolding of the fourth helix on temperature increase tuning the binding affinity of Cnu to H-NS in a temperature dependent manner^32^. Similarly, the N-terminal domain of H-NS is also highly charged^33^ with a charge asymmetry complementary to that of Hha-family members enabling binding^31^. Given that Hha-family members together with H-NS have been implicated in osmosensory response^1,18–20^, it is possible that the asymmetric charge distribution is an evolutionarily selected feature that enables salt-dependent modulation of structure that could be of functional importance.

In this work, we investigate the role of solution osmolarity conditions in modulating the structure, stability, and conformational properties of Cnu through an array of experimental and computational probes. We find that conformational changes within the native ensemble of Cnu (and not between folded and unfolded states) contribute to distinct tertiary structural rearrangements that swell the structure with decreasing ionic strength. The conformational changes in Cnu act synergistically with H-NS structural disorder and oligomerization contributing to two orders of magnitude difference in binding affinity across the temperature and ionic strength. We thus uncover a regulatory mechanism wherein oligomerization of H-NS on Cnu increases continuously with temperature at low ionic strength thus compensating for weakening affinity at higher temperatures. Our findings highlight an intricate interplay between the conformational behavior of a protein pair and thermodynamic environmental variables determining the effective functional output, a feature that could underlie other stress-responsive mechanisms involving these regulatory proteins.

## Results

### Breakdown of structure-stability relationship

The unfolding equilibrium of Cnu is highly reversible (Supplementary Fig. 1) at the chosen representative conditions of low (14.5 mM ionic strength, IS), medium (170 mM IS), and high osmolarity (500 mM IS). Differential scanning calorimetry (DSC) experiments reveal a progressive stabilization of Cnu with increasing ionic strength (Fig. 1c, Supplementary Fig. 2). Electrostatic calculations employing the Tanford-Kirkwood (TK) algorithm^34,35^, however, point to a weakening of native charge–charge interactions with increasing salt (Supplementary Fig. 3A), at odds with experiments. The observed difference between TK model predictions and experiments could arise from two different sources – the dielectric differences assumed in the TK model do not hold true for Cnu given the large number of charged residues in the protein, or, the native ensemble of Cnu is itself modulated with salt thus contributing to erroneous predictions.

To explore the extent of structural modulation (if any), we performed far-UV CD experiments at a range of ionic strength in addition to the three representative conditions to probe for generality (Fig. 1d, e). Increasing salt stabilizes the structure thus increasing the melting temperature (*T*_m_), in agreement with DSC measurements (Supplementary Fig. 4A). On the other hand, we observe an unexpected anti-correlation between structure and stability from far-UV CD measurements; this is apparent in the plot of the far-UV CD signal at, say, 25 °C versus ionic strength (Fig. 1e). While structural stability increases with salt, the secondary structure content decreases, highlighting a breakdown of structure-stability relationship largely observed to hold true in well-folded single domain proteins. Furthermore, the tertiary structural signatures of Cnu exhibit dramatic differences with ionic strength encompassing both tryptophan and tyrosine spectral regions of near UV CD spectra (Fig. 1f) with non-trivial changes in the melting profiles (Supplementary Fig. 4B).

### Native ensemble heterogeneity

**Fig. 2:**
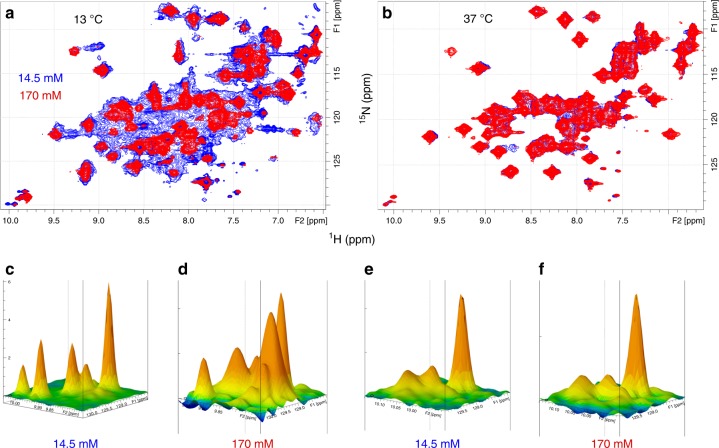
Salt-temperature tunable structural heterogeneity. ^1^H-^15^N HMQC spectra of Cnu at the indicated ionic strength conditions and temperatures 13 °C (**a**, **c**, **d**) and 37 °C (**b**, **e**, **f**). The panels in the bottom row represent the multiple resonances observed for the indole moiety of W67 located in the fourth helix

It is possible (though unlikely) that the observed differences in far- and near-UV CD spectra could also arise from an altered native structure with the extent of heterogeneity remaining unaffected. To probe for this, we acquired ^1^H-^15^N HMQC spectra at low and medium IS (14.5 and 170 mM) and at two temperatures (13 °C and 37 °C). The ensemble at 170 mM IS and 13 °C is heterogeneous with multiple conformations in slow exchange within the native ensemble (note the cluttered resonances in red in Fig. 2a). Lowering the ionic strength enhances the heterogeneity with the appearance of additional broadened peaks (blue in Fig. 2a). Interestingly, the spectra are similarly broadened at 37 °C indicating that the effect of salt can be counteracted by temperature (Fig. 2b).

The observations above preclude the possibility of a single structure and hence a detailed resonance assignment. However, since the tryptophan indole region of a typical HMQC spectrum is well separated from the rest (>9 ppm in the proton direction and >120 ppm in ^15^N), it is possible to probe for structural heterogeneity arising from the fourth helix of Cnu where the sole tryptophan (W67) is located. In agreement with previous observations^32^, several resonances are observed for W67 that are modulated both as a function of salt and temperature (Fig. 2c–f). The indole resonances are well separated and relatively sharp at low temperatures and at both ionic strength conditions, but they broaden and merge as the temperature increases. However, the number, intensity and position of the individual indole resonances are distinct between the two ionic strength conditions at low temperature highlighting that the structural ensemble is quite different even at the lowest temperature, corroborating near-UV CD observations. The distinct resonances corresponding to the indole moiety are indicative of a conformational exchange time-scale that is slower than 8 milliseconds^32^.

### Structural swelling with decreasing ionic strength

**Fig. 3:**
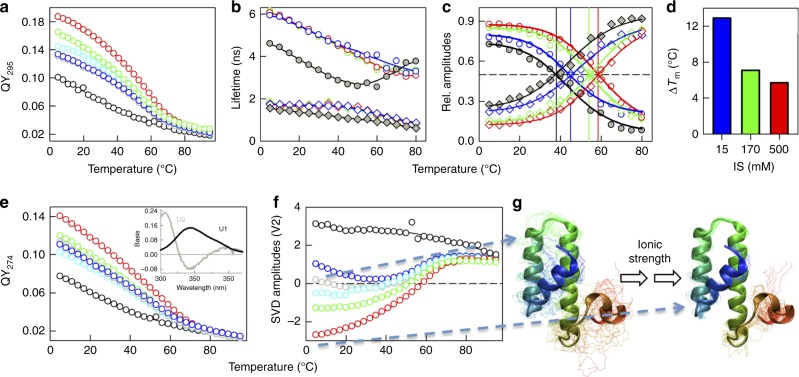
Large-scale structural re-arrangements within the native ensemble. **a** QY of W67 as a function of temperature at 14.5 mM (blue), 43 mM (gray), 90 mM (cyan), 170 mM (green), and 500 mM IS (red). The QY at 6 M urea is shown as a reference (black). Note the large changes in QY even at the lowest temperatures where unfolded population is negligible. **b** Longer and shorter fluorescence lifetimes of W67 as circles and diamonds, respectively. The color code is the same as in panel **a**. The filled gray symbols represent the life-times of tryptophan in the reference peptide C-pep. **c** The relative amplitudes following the same color code as in **b**. The vertical lines indicate the inflection points. **d** Difference in melting temperatures between far-UV CD and W67 life-time amplitudes calculated as *T*_m_ (CD) – *T*_m_ (Life-time). **e** QY of the Cnu upon excitation at 274 nm exhibiting similar characteristics as that of QY_295_ and following the same color code as in panel **a**. (Inset) Spectral deconvolution of temperature-wavelength data shows two components, the first of which accounts for the average spectrum (U1) and the second represents an anti-correlation between tryptophan and tyrosine fluorescence emission bands (U2). **f** The amplitude of U2 following the color code as in panel **a**. **g** A pictorial representation of the conformational events monitored by the data shown in panel **f** with a large structural heterogeneity at low IS that decreases continuously with increase in the salt concentration. Source data are provided as a Source Data file

The conformational behavior of the fourth helix can be directly monitored through the fluorescence properties of W67. In this regard, the W67 QY (exciting at 295 nm) decreases continuously with decreasing ionic strength (Fig. 3a) with the QY approaching that of the fully unfolded protein at 6 M urea (black in Fig. 3a). This suggests that the fourth helix unpacks thermodynamically earlier from the rest of the structure with decreasing ionic strength; this is expected to result in an increasingly larger melting temperature difference between global (far-UV CD or DSC) and local probes (QY). However, estimating melting temperatures from QY data is challenging as intrinsic changes in fluorescence and conformational changes are coupled.

To overcome this limitation, we measured the fluorescence lifetimes of W67 as a function of temperature and ionic strength as such experiments directly decouple intrinsic signals (number of life-times and their magnitude) from the populations (amplitudes). We find that W67 exhibits two lifetimes (fluorescence life-time decays best described by double-exponential fits, Supplementary Fig. 5): a longer lifetime corresponding to a folded-like ensemble and a shorter lifetime representing the unfolded ensemble (Fig. 3b). A reference peptide corresponding to the sequence of the fourth helix, C-pep, displays a very different lifetime and amplitude dependence, clearly indicating that the differences we observe in the protein arise from non-local interactions of W67 (filled gray in Fig. 3b, c). Interestingly, the protein W67 lifetimes are themselves unaffected by ionic strength but the amplitudes of the longer lifetime decrease with temperature signaling a decrease in the population of states monitored by this component (Fig. 3c). The inflection point of this curve is more than 12 °C lower than far-UV CD estimates at 14.5 mM while becoming more similar (but still distinct) at 170 and 500 mM ionic strength conditions (Fig. 3d). These experiments demonstrate that the fourth helix detaches from the rest of the structure at lower temperatures with the maximal differences at 14.5 mM ionic strength in agreement with increased conformational heterogeneity observed from NMR (Fig. 2).

The increased structural heterogeneity with decreasing salt should lead to a concomitant structural swelling or an increased hydrodynamic volume. In fact, structural modulations of Cnu with temperature, urea, and mutation all reveal a proportionate increase in hydrodynamic volume demonstrated earlier through a combination of size-exclusion chromatography (SEC) and analytical ultracentrifugation (AUC) techniques^32,36,37^. However, it is challenging to discern the same at low ionic strength employing SEC^38^, light scattering^39^, or AUC^40^ due to non-specific interactions with the column matrix, strong inter-particle interactions, and primary charge effects, respectively. On the other hand, we had earlier shown that an increase in hydrodynamic volume is accompanied by an increase in distance between a natural intra-molecular tyrosine–tryptophan donor–acceptor pair^37^. Y40 (located in the third helix) and W67 (fourth helix) lie within 12 Å of each other (*r*_0_ for this pair) providing a direct avenue to monitor relative distances with ionic strength.

The QY of Cnu upon excitation at 274 nm exhibits similar ionic-strength dependence as the 295 nm excitation (Fig. 3e). Spectral deconvolution of the wavelength-temperature-salt emission spectra by singular value decomposition (SVD) reveals two components, with the first representing the average spectrum (U1 in the inset to Fig. 3e, Supplementary Fig. 6) and the second highlighting an anti-correlation between the tyrosine and tryptophan fluorescence emission bands at ~305 and ~350 nm, respectively (U2 in the inset to Fig. 3e). The amplitude of the second basis spectrum accounts for changes in relative distance between Y40 and W67. Specifically, at low temperature and high ionic strength, the amplitude is more negative indicating a larger W67 emission (hence higher FRET due to shorter Y40-W67 distance; Fig. 3f, g) but at lower ionic strength the amplitude becomes less negative indicating larger Y40 emission (hence lower FRET due to a longer Y40-W67 distance; Fig. 3f, g). At 6 M urea, conditions wherein the protein is completely unfolded, the amplitude is significantly higher in magnitude compared to other solvent conditions. This comparison clearly indicates that the native ensemble at low ionic strength approaches that of the 6 M urea in terms of the Y40-W67 distance. It is possible that the Y69-W67 distances (within the 4th helix and separated by one residue) are also modulated with salt, but the changes in distance are expected to be just 2.4 Å even on complete unfolding (from a Freely-Jointed Chain model for the unfolded state, Supplementary Fig. 7). Specifically, Y69-W67 distances (in both folded and unfolded cases) are shorter than the Förster radius *r*_0_ for the W-Y pair (12 Å) and therefore the corresponding contribution to FRET change is expected to be small, whereas the Y40-W67 distance in the folded state is 11 Å (close to *r*_0_) and therefore any change in this distance should make a much bigger contribution to the observed FRET change, apart from minor contributions arising from two other tyrosine residues (Y23 and Y28 at 19.6 and 27.2 Å from W67) that are located in the second helix (Fig. 1a).

**Fig. 4:**
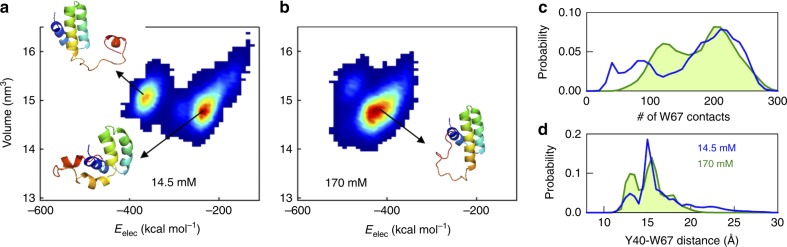
Evidence for structural swelling from simulations. **a, b** Two-dimensional probability densitites derived from REMC simulations at 39 °C plotted in the spectral scale (blue to red represent low to high probabilities) at 14.5 mM (**a**) and 170 mM IS (**b**). The ordinate represents the protein volume and the abscissa is a measure of the electrostatic interaction energy that splits the native ensemble into two sub-ensembles at 14.5 mM IS. **c**, **d** Distribution of W67 contact probabilties (**c**) and Y40-W67 C_α_-C_α_ distances (**d**). Source data are provided as a Source Data file

To further validate these observations we performed implicit-solvent replica-exchange Monte-Carlo simulations at two representative IS conditions of 14.5 and 170 mM (see Methods). At 14.5 mM and 39 °C, the native ensemble of Cnu is composed of two-subpopulations – one in which the structure is compact but with extensive disorder in the 4th helix and the other corresponding to an expanded globule in which the overall three-dimensional shape is significantly perturbed (Fig. 4a). Though the overall electrostatic interaction energy is favorable, such large-scale structural modulation is potentially a result of local electrostatic frustration (Supplementary Fig. 2B). These results are in agreement with NMR spectra that point to extensive structural heterogeneity at low IS compared to the higher IS conditions. The ensemble at 170 mM is predominantly well folded but with significant structural disorder in the 4th helix (Fig. 4b). The tryptophan contact probability and Y40-W67 distance distributions show larger variability at 14.5 mM compared to 170 mM IS (Fig. 4c, d) in agreement with the steady-state and time-resolved fluorescence measurements. The smaller volume changes are likely a result of force-field effects^41^ that are known to promote compact and collapsed structures even on partial unfolding. The ensembles identified from simulations therefore represent just the lower bound on the conformational heterogeneity of Cnu.

### Tunable binding cooperativity

**Fig. 5:**
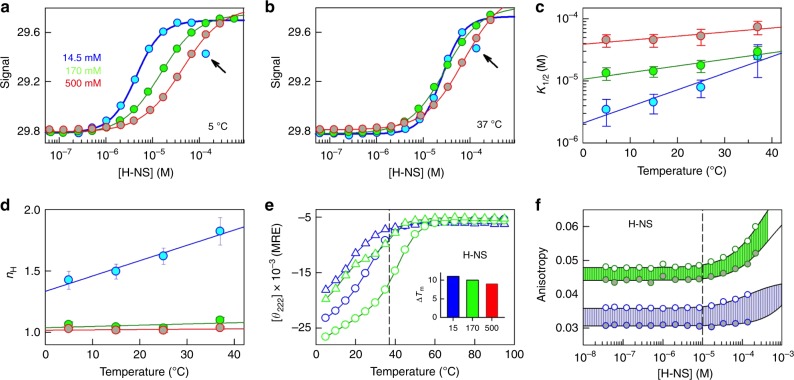
Switching binding cooperativity upon ionic strength modulations. Blue, green, and red represent 14.5, 170, and 500 mM ionic strength, respectively. **a**, **b** Binding isotherms at 5 °C (**a**) and 37 °C (**b**). The binding reaction was followed by the intensity averaged wavenumber represented as Signal in the ordinate (see Methods). Note that there is a roll-over at high H-NS concentrations (arrow). The curves are fit to Hill binding equations. **c**, **d** The mid-point of binding in units of dissociation constant (*K*_1/2_; **c**) and Hill coefficients (*n*_H_; **d**). The lines through the points are shown to guide the eye. **e** Far-UV CD monitored thermal unfolding curves of H-NS at concentrations of 6 μM (triangles) and 18 μM (circles) at 14.5 (blue) and 170 mM IS (green). Note that H-NS is fully unfolded at 37 °C at both the ionic strength conditions and at low protein concentrations (dashed line). (Inset) The difference in melting temperatures between the two H-NS concentrations at the three ionic strength conditions calculated as *T*_m_ (18 μM) – *T*_m_ (6 μM). **f** Aniostropy titration curves of tryptophan labeled H-NS with tyrosine labeled H-NS to identify the minimum concentration at which H-NS starts to self-oligomerize. It can be seen that there is negligible oligomerization at 14.5 mM (blue) compared to 170 mM IS (green, upshifted by 0.01 units for visual representation). The open and filled gray circles represent 5 °C and 37 °C, respectively. The curves are a fit to 1:1 binding equilibrium. The vertical dashed line signals a minimum oligomerization concentration of 10 μM in the absence of Cnu (more clearly seen at 170 mM IS). Error bars show 68% confidence intervals. Source data are provided as a Source Data file

What is the functional consequence of such large-scale but salt-tunable structural heterogeneity in Cnu? The temperature-osmolarity stress-responsive mechanisms in enterobacteriaceae involve interactions between Cnu and the transcription repressor H-NS apart from DNA^23,31^. The binding interface of Cnu involves precisely those residues in the fourth helix (and the associated regions in the third helix; Fig. 1a) that display complex conformational behavior with temperature and salt as observed for all members of the Hha-family^23,31,32,37^. Titration experiments reveal that the binding of H-NS (residues 1–59 of the N-terminal domain, see Methods and Supplementary Fig. 8A) to Cnu can be adequately described by an apparent 1:1 binding equilibrium (Hill coefficient *n*_H_~1) at all temperatures and at 170 and 500 mM ionic strength (green and red in Fig. 5a–d). The stability of the complex weakens with increasing temperature under these ionic strength conditions.

The binding isotherms are however noticeably sharper at 14.5 mM IS indicating the formation of a higher-order complex (blue in Fig. 5a–d). Quantitative analysis of the binding isotherms employing the Hill equation reveals an increase in the Hill coefficient (from 1.4 ± 0.07 to 1.8 ± 0.11) with increasing temperatures but with the apparent binding affinity weakening by an order of magnitude. At high H-NS concentrations and at all temperatures, the signal decreases with increasing concentration of H-NS suggestive of competitive formation of H-NS multimers (dimers, trimers etc^22,42,43^.) at the expense of Cnu:H-NS complex (arrows in Fig. 5a, b). The fractional Hill coefficients potentially arise from an ensemble of molecular species in solution^44^ – Cnu:H-NS, Cnu:(H-NS)_2_ etc. - with their relative populations determining the sharpness of the binding isotherm and hence the average Hill coefficient.

The switch in binding cooperativity and the decrease in signal at high H-NS concentrations are a manifestation of the equally intricate conformational behavior of H-NS in solution. H-NS by itself displays steep unfolding curves and concentration-dependent melting temperatures indicative of sensitivity to salt, temperature, and concentration (Fig. 5e and Supplementary Fig. 8B, C). Interestingly, H-NS exhibits partial order–disorder equilibrium between 5 °C and 37 °C at the two explored protein concentrations at 14.5 mM IS (6 and 18 μM); a similar equilibrium is also observed at 170 mM IS but only at low protein concentrations. Anisotropy titration profiles of H-NS with itself (one labeled with tryptophan in the C-terminal tail and the other without it, see Methods) indicates dimer or high molecularity multimer formation at concentrations >10 μM explaining the differences in melting temperatures from far-UV CD experiments (Fig. 5f). Moreover, at 14.5 mM and 37 °C and in the absence of Cnu, there is little evidence for higher-order oligomer formation due to the completely unfolded nature of the H-NS ensemble under these conditions (note the flat anisotropy titration profile in blue, Fig. 5f). Fitting the anisotropy profiles to monomer-dimer equilibrium (the simplest equilibrium model) results in an apparent *K*_D_ ~100–150 μM. It thus appears that the enhanced structural heterogeneity of both Cnu (through structural redistributions within the native ensemble) and H-NS (through unfolding) at low IS act synergistically to seed the formation of an ensemble of heteromers (*n*_H_ > 1), with previous formation of Cnu:(H-NS)_x_ complexes facilitating H-NS oligomerization. At higher H-NS concentrations and low IS, H-NS oligomers (homomers) are preferentially formed over Cnu:H-NS complexes contributing to the observed decrease in signal. At 170 mM IS, H-NS preferentially binds Cnu (heteromer is preferred over homomer) following a 1:1 binding model with no signs of decrease in binding signal, at least in the concentrations range explored in the current work.

The experimental 500 mM IS corresponds to osmolarity conditions in the gut of a mammal, signaling successful invasion; under these conditions, the binding affinity of Cnu to H-NS is very weak (*K*_1/2_ approaching ~100 μM at 310 K) promoting the expression of virulence genes (as stronger Cnu-H-NS interaction suppresses expression). However, at medium IS (170 mM), the affinity increases by a factor of ~4 contributing to stronger suppression as expected from the electrostatic nature of the interaction between Cnu and H-NS^31^. Accordingly, at low IS (14.5 mM) the weaker charge screening promotes even stronger interaction between Cnu and H-NS. Such low IS conditions are characteristic of the early stages of osmotic stress response when the external salt concentrations are low thus promoting the inflow of water and cellular swelling^7^. But these conditions are seldom experienced by the bacteria on infection and therefore even if the temperature is increased to 37 °C (body temperature of a mammal) the expression of virulence genes will not provide any selective advantage to the organism. To over-ride this intrinsic temperature effect and to minimize transcription of virulence genes, the conformational characteristics of Cnu and H-NS have been selected to form higher-order molecular complexes (*n*_H_ > 1) for efficient repression. Thus, from a functional viewpoint, the cooperativity effects likely arise from the requirement to enable graded suppression of virulence genes as a function of temperature and solvent ionic strength.

## Discussion

We have demonstrated that Cnu acts as an osmosensor by modulating its structure, local stability, and dimensions continuously in response to solution ionic strength. This unique sensitivity has its origins in the asymmetric distribution of charged residues on the surface of Cnu that keep it at the threshold of local disorder. In other words, Cnu samples a large array of conformations within the native ensemble driven primarily by local electrostatic frustration resulting in a graded response to osmolarity conditions. These features are remarkably matched in the binding partner, H-NS, which displays a similar salt and temperature sensitivity but that also undergoes order–disorder-oligomer equilibrium. Both proteins sample larger conformational space at lower ionic strength conditions (with H-NS completely unfolding at 37 °C and 14.5 mM IS) the combination of which contributes to the formation of higher-order structures.

Switching cooperativity or the populations and identity of different oligomeric species enables a fine-tuned response to environmental variables with the binding affinity of the complex varying by nearly two orders of magnitude across temperature and ionic strength conditions (Fig. 5). At high ionic strength found after successful host invasion, 1:1 Cnu-H-NS complexes seem to be preferentially formed but with very weak affinity thus promoting expression of virulence genes. At low IS, the Cnu affinity for H-NS increases and this is linked to increase repression of virulence genes. According to the current models of H-NS repression, this would imply that Cnu binding in fact enhances H-NS oligomerization. At low ionic strength and even at high temperatures, H-NS oligomerization predominates thus promoting efficient repression of virulence genes. The switch from heteromers (Cnu-H-NS complexes) to homomers (H-NS oligomers) with increasing H-NS concentration provides further evidence for ‘molecular mimicry’ wherein Hha-family members can substitute for H-NS, adding another layer of regulation^45^. Moreover, there could be a difference in the heteromer/homomer equilibrium and molecularity depending on the identity of the Hha-family member; particularly, it has been shown before that an ensemble of oligomeric species constitute Hha: H-NS solutions at high H-NS concentrations (~100 μM or higher^31,46^) and intermediate ionic strength conditions (170 mM or higher), highlighting a certain level of context-dependence driven by specific sequence differences. Interestingly, Hha is more abundant than Cnu under intermediate osmolarity conditions with the latter being overexpressed only when Hha is depleted^47^. The Cnu mediated osmolarity response could also involve StpA, an H-NS paralog, as they have been shown to form heteromeric complexes^47^. Taken together with our study, these observations point to a role of not just Cnu:H-NS but also Hha:StpA and Cnu:StpA complexes, their relative abundance, and the conformational behavior of Hha contributing to the robustness of osmolarity response in enterobacteria, facets that demand a detailed study similar to that presented here.

Hha-like proteins and H-NS are involved in numerous pathways implicated in stress-responsive mechanisms including changes in temperature, salt, and pH. In this regard, we have previously shown that the fourth helix of Cnu exhibits graded structural polymorphism and hence functions as a thermosensor^32^. On the other hand, the protonation of a partially buried histidine (H45V) in Cnu makes its structure sensitive to the pH range 3–7^36^. The underlying molecular mechanism in all these cases involves the disruption or formation of the binding interface with H-NS through protonation of H45 (pH response), electrostatic frustration (osmolarity response), and graded local stability of the fourth helix (temperature response). Cnu thus serves as an interesting system where energetic frustration is engineered locally into the structure intricately linking the degree of local folding, stability, and function. The role of disorder and its effect on cooperativity have been well studied from the perspective of disordered proteins with interesting mechanistic outcomes due to large contributions from conformational entropy apart from specific interactions^48–52^. Our work on Cnu extends these observations to partial disorder even within the native ensemble that could have regulatory consequences. Similar native ensemble dynamics have also been reported in Hha^23^ and YmoA^31^, homologs of Cnu, underscoring the fact that such structural motions are conserved across members of the Hha-family. It is therefore as important to understand the extent of structural heterogeneity in the native ensemble of proteins that could pave the way for designing rational mutations that modulate native dynamics and populations, and hence the functional output. Specifically, mutations in Cnu that enhance or eliminate osmo- and thermo-sensory responses could provide a fundamental understanding of how such stress-responsive features are imprinted at a molecular level, an aspect we are currently working on.

## Methods

### Cnu expression

Cnu was expressed and purified following the protocol detailed before^32^. The construct has four additional residues at the N- and C-terminus with sequences KEKE and EKEK, respectively. This well-mixed sequence is added to promote solubility as the protein construct without the tail residues goes primarily into inclusion bodies^32^. All experiments reported in this work were carried out at pH 8.0 in sodium phosphate buffer. Sodium chloride was added in appropriate amounts to prepare different ionic strength buffer solutions.

### H-NS purification

H-NS_1–59_ gene corresponding to the protein sequence (the tyrosine variant) -MSEALKILNNIRTLRAQARESTLETLEEMLEKLEVVVNERREEESAAAAEVEERTRKLY - was cloned into NdeI and SpeI restriction sites of pTXB1 vector (New England Biolabs) and transformed into *E. coli* BL21(DE3) cells. A single transformed colony was used to inoculate 2L LB media containing ampicillin. The culture was induced with 1 mM IPTG on reaching OD~1 and grown for 8 h at 37 °C. The cells were lysed by sonication, cell debris removed by centrifugation and the clear lysate was passed through a 5-ml chitin beads column and subsequently washed with 20 column volume (CV) of cell lysis buffer (20 mM HEPES pH 8.5, 500 mM NaCl, 1 mM EDTA). The cleavage reaction was initiated by passing 5 CV of cleavage buffer (lysis buffer + 50 mM β-ME), and the column was incubated at 23 °C for 30 h. The cleaved product was lyophilized, dissolved in milli-Q water, and then loaded onto a HiLoad Superdex 75 pg (GE, 320 ml CV) size-exclusion column with 150 mM sodium acetate buffer (pH 8.0) as the mobile phase. The fractions containing HNS_1–59_ were pooled together and lyophilized. A complete list of primers used is provided in Supplementary Table 1.

### Differential scanning calorimetry

The apparent heat capacity of Cnu was measured in Microcal VP-DSC (Malvern, UK) at protein concentrations ranging from ~40 to ~120 µM at a scan-rate of 60 °C/hr. The protein samples were desalted and degassed prior to loading in to the calorimetric cell. Several buffer–buffer scans were recorded before and after running the protein sample to ensure baseline reproducibility. The apparent heat capacity was converted to absolute units following the method of Sanchez-Ruiz and coworkers^53^.

### Circular dichroism

Far- and near-UV CD spectra were collected in a Jasco J-815 spectrophotometer coupled to a Peltier unit employing quartz cuvettes of pathlengths 1 and 10 mm, respectively. The signals are reported in conventional mean residue ellipticity (MRE) units of deg. cm^2^ dmol^−1^.

### Nuclear magnetic resonance

^1^H-^15^N HSQC, SOFAST-HMQC, and TROSY spectra were recorded in a ^15^N-labeled sample of Cnu on Bruker Avance III 600 and 800 MHz spectrometers with cryogenic probes as detailed before^32^. The protein concentrations were ~80 µM for 14.5 and 170 mM ionic strength experiments. Spectra were not acquired at 500 mM ionic strength due to poor signal-to-noise ratio that accompanies solutions with large salt concentrations in a standard 5 mm tube.

### Steady-state fluorescence and quantum yield measurements

The fluorescence emission spectra of Cnu were recorded in a Chirascan™-Plus qCD instrument coupled to a Peltier unit (Quantum Northwest Inc.). The protein sample was excited at 274 nm using a Xenon lamp and the emission spectra were recorded in the wavelength range of 280–550 nm. Temperature-dependent emission spectra were recorded at an interval of 3 °C from 5 to 95 °C. W67 in Cnu was selectively excited at 295 nm and the emission spectra were recorded from 300 to 550 nm. The quantum yields (QY) of the protein on excitation at 274 nm (QY_274_) or W67 on 295 nm excitation (QY_295_) were estimated using NATA as a reference (0.13 in water at 25 °C).

### Time-resolved fluorescence

The time-dependent fluorescence intensity decay of the sole tryptophan in Cnu (W67) was recorded in a ChronosBH (ISS Inc.) spectrometer. The samples were excited with 300 nm LED. The excitation pulse and emitted photons were passed through UV grade Glan-Thompson polarizers set at 0° and 54.7°, respectively, from the vertical *z*-axis. The instrument response function (IRF) was measured using Ludox solution. The emitted photons were passed through a 345-nm long-pass filter (SCHOTT) to minimize scattering artifacts. All decay curves were recorded until the peak count reached 10^4^ or the total count approached 10^8^. The traces were best reproduced by bi-exponential functions with the chi-square values being <1.5 at all temperatures and solvent conditions.

### Binding studies

The binding of H-NS to Cnu results in a blue shift in the fluorescence emission spectra of Cnu W67 upon excitation at 295 nm. Following preliminary experiments, the Cnu-H-NS binding was directly monitored by collecting the fluorescence emission spectra of ~2 µM Cnu at different concentrations of H-NS (~30 nM–~140 µM final concentration). This concentration was chosen to avoid inner-filter effects that can accompany higher protein concentrations. The excitation and emission bandwidths were set to 5 and 10 nm, respectively. The emission spectra were collected following a uniform equilibration time of 5 min at every temperature and ionic strength condition. The binding reaction was followed by the intensity-averaged wavenumber ($\bar \nu _{av}$) defined as

1 $$\bar \nu _{av} = 10^4\;\frac{{\mathop {\sum }\nolimits_\lambda I\left( \lambda \right)\lambda ^{ - 1}}}{{\mathop {\sum }\nolimits_\lambda I\left( \lambda \right)}}$$

where *I*(*λ*) is emission intensity at wavelength *λ*. This accounts for changes in the emission maximum and intensity of W67 fluorescence on H-NS titration.

### H-NS oligomerization

The oligomerization of HNS_1–59_ was monitored by steady-state tryptophan anisotropy. For this study, we generated a variant of H-NS with a tryptophan at its C-terminus, H-NS_W_. The H-NS_W_ (2 µM) samples were mixed with varying concentrations of H-NS (~30 nM–~140 µM, the tyrosine variant), and at each temperature the samples were incubated for five minutes before recording anisotropy. The H-NS_W_ samples were excited at 295 ± 2 nm, and the emitted photons were filtered through a 320-nm cut-off filter.

### Implicit solvent MD simulations

Replica-exchange Monte-Carlo simulations (REMC) were performed employing the CAMPARI package^54^ starting from the fully folded structure of Cnu with temperature-dependent dielectric constant and solvation free-energies^55^. ABSINTH implicit solvent model with OPLS charges and explicit ions were used to simulate different ionic strength conditions. The protein was placed at the center of a spherical shell of diameter 100 Å with explicit counter-ions to neutralize the system. Additional 52 and 428 ion pairs were added to mimic experimental 14.5 mM and 170 mM ionic strength conditions, respectively. The REMC was performed over 22 temperature replicas equally spaced in the range of 280 K to 450 K with exchange attempts every 10^4^ steps (average exchange probability ~0.46). Simulations were run in parallel for 6 × 10^7^ steps per replica and the coordinates were collected every 500 steps. Structural analysis was performed on the snapshots from the final 3 × 10^7^ steps.

### Reporting summary

Further information on research design is available in the Nature Research Reporting Summary linked to this article.

## Supplementary information

Supplementary Information

Reporting Summary

Source Data

## Data Availability

Data supporting the findings of this manuscript are available from the corresponding author upon reasonable request. A reporting summary for this Article is available as a Supplementary Information file. The source data underlying Figs. 1, 3–5, and Supplementary Figs. 1–8 are provided as a Source Data file.
