# Supplementary material for: A binding cooperativity switch driven by synergistic structural swelling of an osmo-regulatory protein pair
Source: Nat Commun. 2019 Apr 30;10:1995. doi: 10.1038/s41467-019-10002-9 (PMC6491433; doi:10.1038/s41467-019-10002-9)
Supplement: Supplementary file 1 — Supplementary Information [file 41467_2019_10002_MOESM1_ESM.pdf]

Supplementary Information

A Binding Cooperativity Switch Driven by Synergistic Structural Swelling of an Osmo-Regulatory Protein Pair

Abhishek Narayan,[1] Soundhararajan Gopi,[1] David Fushman[2]

*& Athi N. Naganathan[1]**

[1]Department of Biotechnology, Bhupat & Jyoti Mehta School of Biosciences, Indian Institute of Technology Madras, Chennai 600036, India.

[2]Center for Biomolecular Structure and Organization, Department of Chemistry & Biochemistry, University of Maryland, College Park, MD 20742, USA.

*Corresponding Author: athi@iitm.ac.in

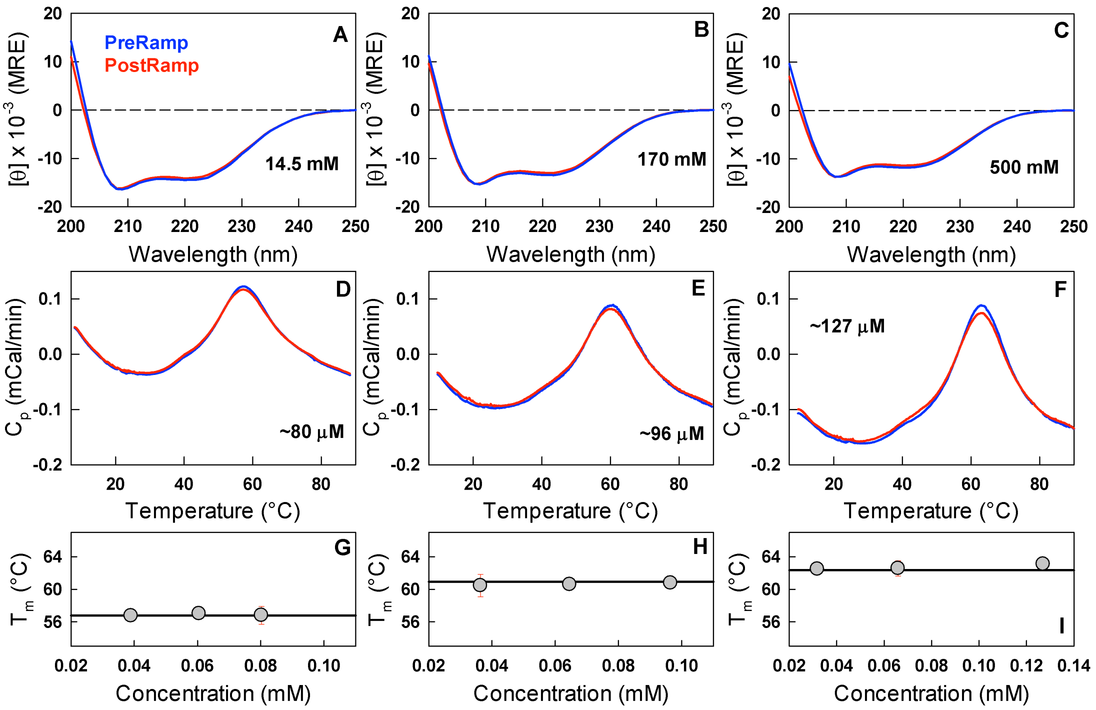

Supplementary Figure 1 The left, middle and right columns represent the experimental data of Cnu at 14.5, 170 and 500 mM ionic strength, respectively. (A-C) Pre- and post-thermal ramp far-UV CD spectra of Cnu at a protein concentration of ~18 μM. (D-F) Raw thermograms of Cnu at the indicated concentrations. The first scan is shown in blue and the second scan of the same sample is shown in red. (G-I) The mean melting temperature from DSC scans at different protein concentrations highlighting large reversibility of Cnu even up till protein concentrations of 127 μM. Error bars represent standard errors of the mean. Source data are provided as a Source Data file.

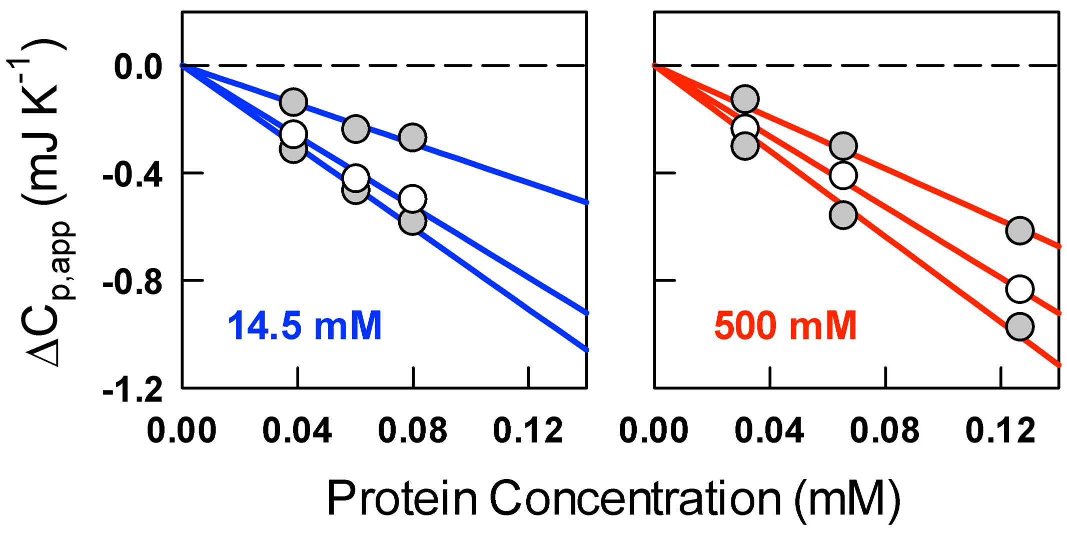

Supplementary Figure 2 Plots of apparent heat capacity as a function of Cnu concentration at temperatures of 9, 27, and 48 °C (in the order of decreasing absolute slopes). The slopes of the lines are employed to calculate the protein absolute heat capacity. Source data are provided as a Source Data file.

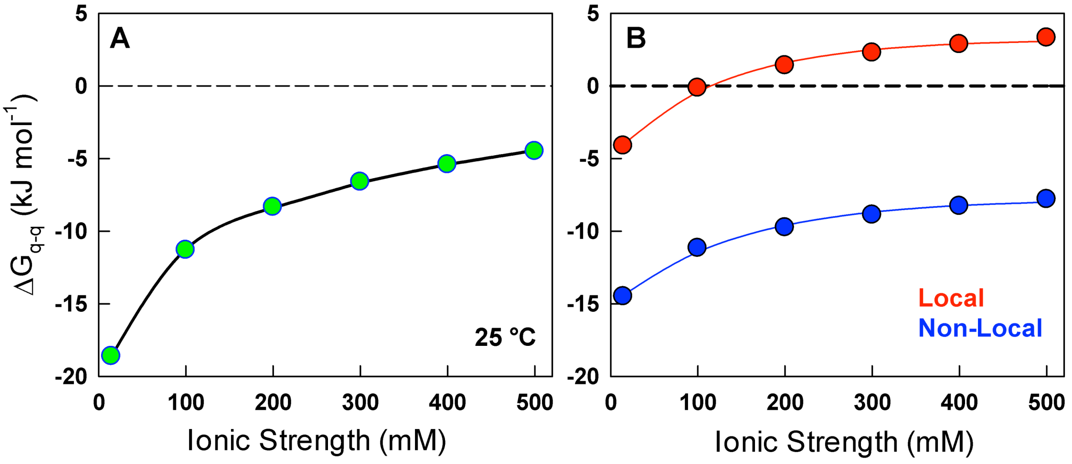

Supplementary Figure 3 (A) Overall charge-charge interaction energy as a function of ionic strength as calculated from the structure of Cnu using the Tanford-Kirkwood algorithm. (B) The same as in panel A but partitioned into local and non-local electrostatic interaction energies. Non-local interactions are identified as interactions between residues with a sequence separation of >4. Source data are provided as a Source Data file.

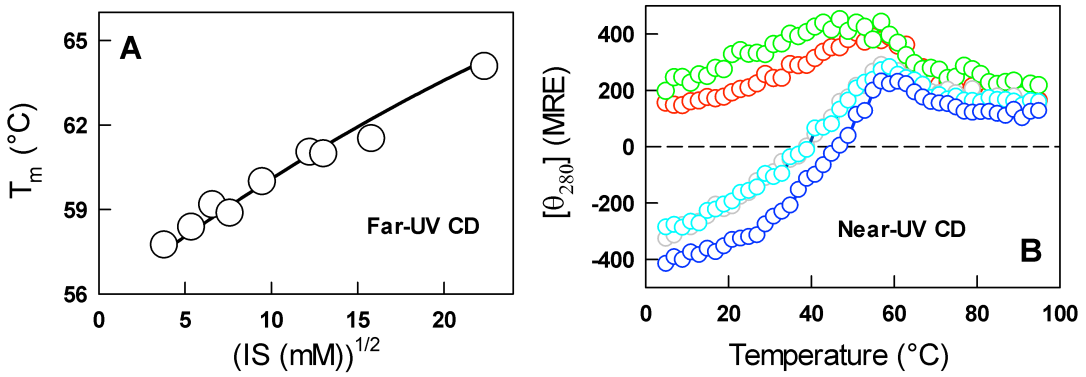

Supplementary Figure 4 (A) The dependence of melting temperatures on the ionic strength (IS) of buffer, from far-UV CD experiments. (B) Thermal unfolding profiles of Cnu monitored by near-UV CD at ionic strength conditions of 14.5 (blue), 43 (gray), 90 (cyan), 170 (green), and 500 mM (red). Source data are provided as a Source Data file.

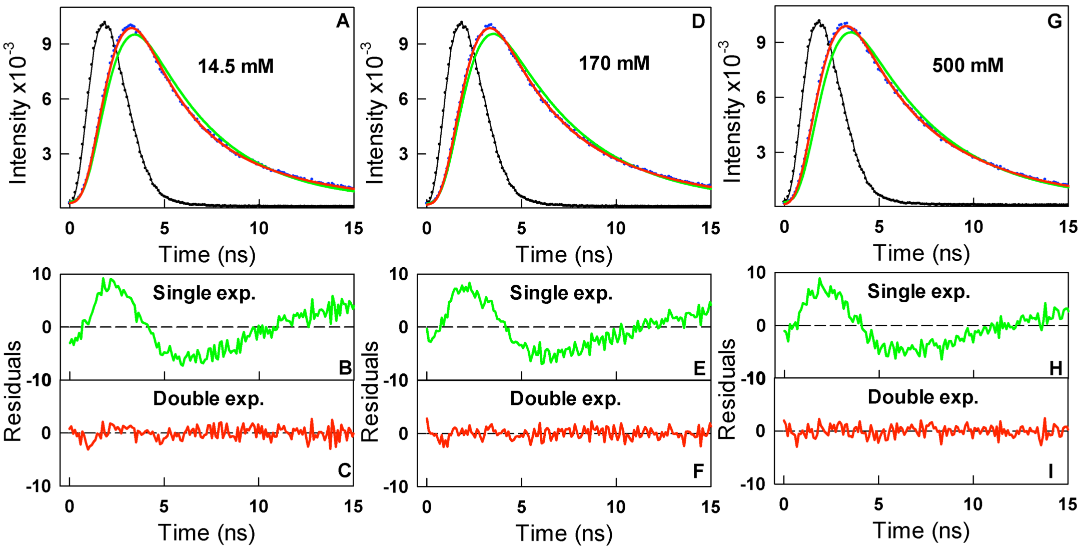

Supplementary Figure 5 The left, middle and right columns plot time-resolved data of Cnu/fit/residuals at 14.5, 170 and 500 mM ionic strength, respectively. (A, D, G) Representative examples of fluorescece life-time decays (circles), fits from single- and double-exponential functions (green and red) and the instrument response function (IRF, black) for Cnu at the indicated ionic strengths and at 25 °C. (B, E, H) Residuals from single-exponential fits to the fluorescence decay curves. (C, F, I) Residuals from double-exponential fits to the fluorescence decay curves. Source data are provided as a Source Data file.

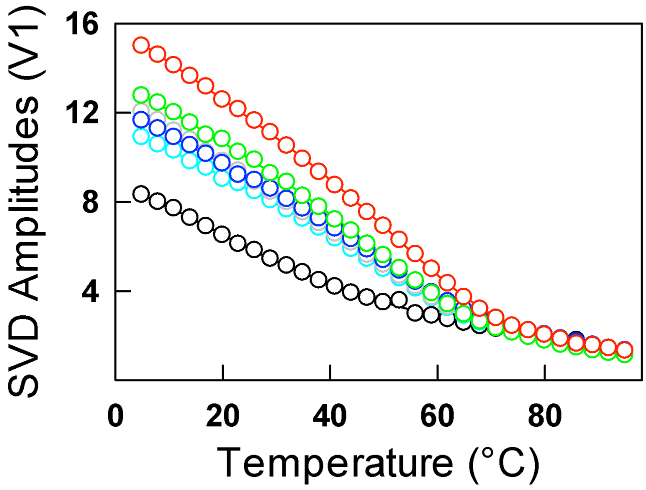

Supplementary Figure 6 The amplitude of the first SVD component (U1, inset in Figure 3D) at varying ionic strength conditions: 14.5 (blue), 43 (gray), 90 (cyan), 170 (green), 500 mM (red), and 6 M urea (black). Source data are provided as a Source Data file.

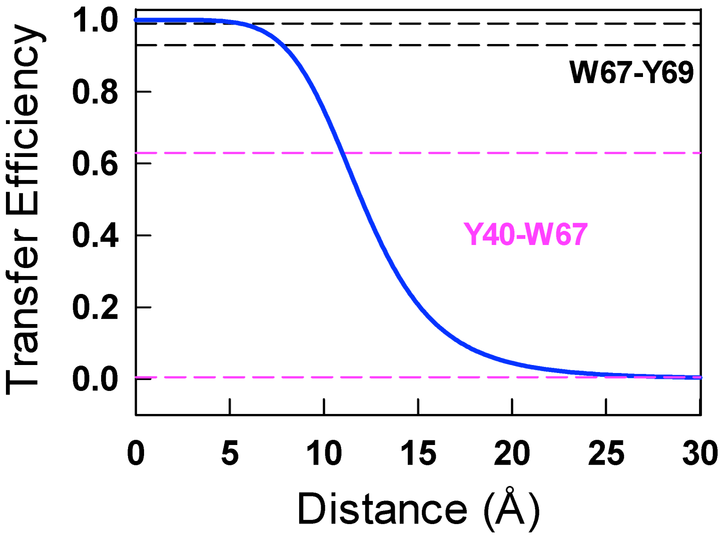

Supplementary Figure 7 The blue curve is the expected dependency of FRET on distance between the donor-acceptor Y-W pair that has an r_0 of 12 Å. In the figure, the high FRET efficiency lines for a given donor-acceptor pair is calculated from the distances in the native structure while the lower FRET efficiency lines are from the unfolded state assuming a Freely Jointed Chain model for the unfolded state:

$$\left\langle r_{i,j}^2 \right\rangle = 2l_p b\left|i-j\right|$$

with l_p = 4 Å (persistence length) and b =3.8 Å (segment length). Source data are provided as a Source Data file.

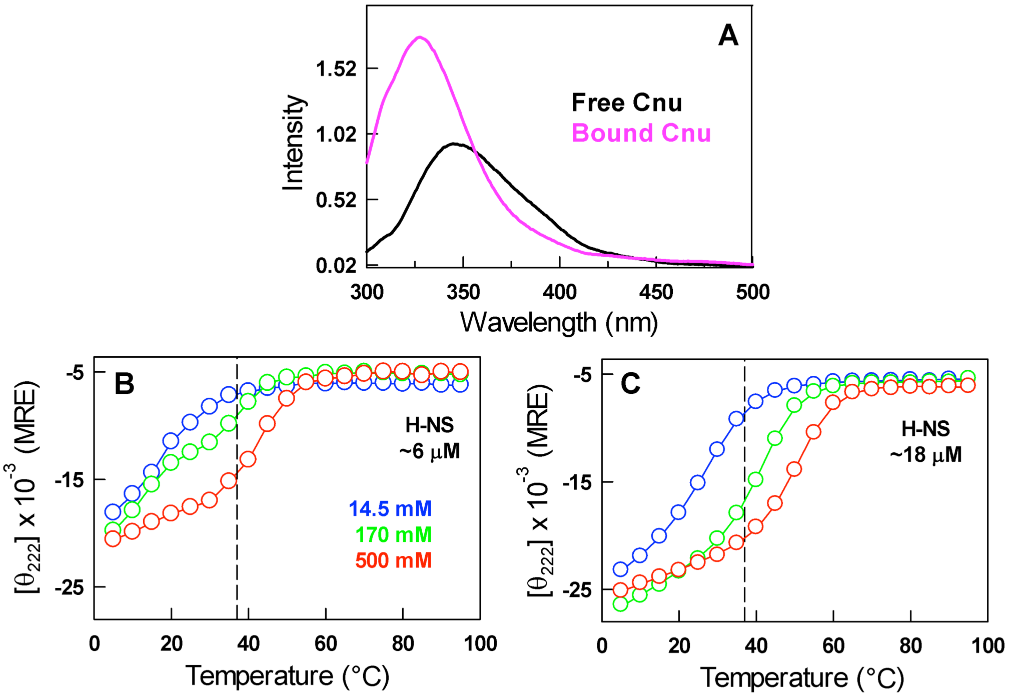

Supplementary Figure 8 (A) A representative example of the fluorescence emission blue shift observed upon titrating Cnu (that harbors the tryptophan) with H-NS (tyrosine variant). The spectra shown in panel A are at 14.5 mM IS, 5 °C and at Cnu:H-NS concentrations of 2 μM: 69 μM. (B-C) Thermal unfolding curves of H-NS monitored by far-UV CD at 222 nm at the two indicated concentrations. The vertical dashed line indicates 37 °C. Source data are provided as a Source Data file.

Supplementary Table 1 List of primers used.

Name	Sequence (5' to 3')
KEKE_Cnu_Forward	GATATACATATGAAAGAAAAAGAAATGACTGTTCA GGACTACTTATTAAAATTTCGC
KEKE_Cnu_Reverse	GATGTAAACTAGTGCATCTCCCGTGATGCATTTTTC TTTTTCTTGGACATAGTGCCAGAC
HNS_W_Forward	CTTTAAGAAGGAGATATACATATGTCCGAAGCCCTG
HNS_W_Reverse	TGGTCAAACTAGTGCATCTCCCGTGATGC
HNS_Y_Forward	GCGCAAACTGTACTGCATCACGG
HNS_Y_Reverse	GTACGTTCTTCGACTTCAG
